# Changes in Regional Homogeneity of Medication-Free Major Depressive Disorder Patients With Different Onset Ages

**DOI:** 10.3389/fpsyt.2021.713614

**Published:** 2021-10-01

**Authors:** Zijian Zhang, Yayun Chen, Wei Wei, Xiao Yang, Yajing Meng, Hua Yu, Wanjun Guo, Qiang Wang, Wei Deng, Tao Li, Xiaohong Ma

**Affiliations:** ^1^Psychiatric Laboratory and Mental Health Center, West China Hospital of Sichuan University, Chengdu, China; ^2^West China Brain Research Center, West China Hospital of Sichuan University, Chengdu, China; ^3^The Fourth People's Hospital of Chengdu, Chengdu, China

**Keywords:** resting-state functional magnetic resonance imaging, regional homogeneity, major depressive disorder, onset-age, hamilton rating scale for depression

## Abstract

**Background:** Neurobiological mechanisms underlying the development of major depressive disorder (MDD) may differ depending on onset ages. Our aim was to determine whether regional homogeneity (ReHo) changes in early-onset depression (EOD) and late-onset depression (LOD) are different, which could also delineate EOD and LOD.

**Methods:** Ninety-one MDD patients and 115 healthy controls (HCs) were recruited, and resting-state functional magnetic resonance imaging data were collected. The ReHo comparison was conducted using analysis of variance.

**Results:** Compared with HCs, MDD patients showed decreased ReHo in the left precentral gyrus and the left middle cingulum area, and increased ReHo in the left middle orbital frontal gyrus and superior temporal gyrus. Compared with LOD patients, young HC separately, EOD patients had significantly increased ReHo in the right inferior frontal triangular gyrus and the left postcentral gyrus. However, compared with young HC, EOD patients showed decreased ReHo in the right superior frontal gyrus/supplementary motor area and the right medial frontal gyrus. ReHo in the right inferior frontal triangular gyrus was negatively correlated with the severity of cognitive disturbance in LOD patients (*r* = −0.47, *p* = 0.002), but not in EOD patients (*r* = 0.21, *p* = 0.178).

**Conclusion:** MDD patients with different onset ages may have different pathophysiological mechanisms; the EOD patients had more abnormal ReHo than LOD patients in the prefrontal lobe, especially the right inferior frontal triangular gyrus.

## Introduction

Major depressive disorder (MDD) is one of the most frequent psychiatric disorders with a lifetime prevalence of 15–30%, which is mainly characterized by depressed mood, decreased interest, lack of pleasure, slow thinking, worthlessness or guilt, reduced energy, impaired cognition, vegetative symptoms, and suicidal tendency (1–3). The current classification of MDD essentially relies on the presence of a defined number of clinical features and a time period over which these symptoms need to be present, as well as by identification of social function impairment (4, 5). MDD is a highly heterogeneous disorder, and there is a lack of consistent genetic and neurobiological biomarkers that can validate MDD as a distinct or even clear diagnostic entity (6, 7). Furthermore, heterogeneity of clinical presentations and individual differences make it difficult for us to deduce standardized and effective treatment strategies for MDD patients (8). Therefore, understanding the depressive subtypes is important to clarify the pathogenesis of MDD and further guide clinical treatment.

As mentioned above, clinical features of MDD patients during disease episode may vary largely. For instance, symptoms and characteristics of adults with early-onset depression (EOD) differ from those with late-onset depression (LOD) (9–11). EOD patients are associated with more suicide attempts and thoughts, sadness, irritability, diminished concentration, more psychiatric symptoms, agitation, more anxiety, neuroticism, atypical symptoms, higher prevalence of comorbid personality disorders, and higher prevalence of stressful life events preceding onset than LOD patients (10, 12–15). Several studies have indicated that EOD patients have poor outcomes, including unfavorable treatment response, longer duration of symptoms, greater illness severity, higher relapse rate (10, 11, 16, 17), and greater duration of illness, after adjusting for current age (12).

Furthermore, there seems to be a trend that MDD patients with earlier onset age may be more strongly affected by genetic factors (18–25). For example, a research from Sequenced Treatment Alternative to Relieving Depression (STAR ^*^ D) reported that MDD patients with positive family history for depression have a younger onset age than those without (26). In addition, the heritability of MDD is about 37% (27), and the heritability of EOD (<30 years) is higher than that of LOD (>30 years) (47 vs. 10%) (28). Tozzi et al. (19) reported that patients with onset age after 50 years old are not associated with a family history of MDD. A meta-review indicated that the different onset-age depression might be considered as different subtypes of depression (29). Taken together, the neurobiological mechanisms underlying the development of MDD in different onset age may vary. However, definitions of EOD and LOD in the literature have been inconsistent in their age cutoffs.

The median onset age for mood disorders is between 25 and 32 years (30). The cutoff age to differentiate EOD and LOD differs between studies and varies between 20 and 60 years old (9–11, 13, 15, 19, 31–34). A comparison of studies will result in inconsistent findings. Considering the role of different onset age in the pathology of MDD, it is important to define the age cutoff between EOD and LOD according to a biologically based criterium. Therefore, we chose a first-onset cutoff of 25 years, which is associated with family history of MDD (35, 36), and has been used as a cutoff in previous research on mood disorders (15, 34, 37–39) and genetic association studies (40). However, little is known about the neural mechanism underlying the difference between EOD and LOD subtypes.

For decades, neuroimaging has been widely used to explore the neuro- and pathomechanism of MDD. Regional homogeneity (ReHo) has been shown to be an effective index to measure spontaneous neural activity in resting-state functional magnetic resonance imaging (rs-fMRI) analysis (41). ReHo reflects the local neural activity coherence, which measures the homogeneity of the time course in a voxel and its nearest neighbors. Numerous studies have explored spontaneous brain activity in MDD using the ReHo method and provided some evidence of brain abnormalities, of which the abnormal ReHo in frontal cortex was most commonly been reported (42–46). Nevertheless, systematically collected neuroimaging data of medication-free EOD and LOD are still limited (39, 47–49). The regional neural activity difference between EOD and LOD remains unclear.

The aim of the current study was to explore the onset-age differences in the phenotypes of depression including clinical symptoms, brain abnormalities, and the relationship between these two aspects. Based on existing literatures, we hypothesized that (1) the MDD patients have decreased or increased regional neural activity homogeneity compared to HCs, especially in frontal lobe; (2) EOD patients may show more severe disturbance not only in clinical symptoms, but also in ReHo than LOD patients; and (3) specific brain alterations of EOD and LOD patients may be associated with different clinical symptoms.

## Materials and Methods

### Participants

This study recruited 206 right-handed participants including 91 patients with MDD and 115 gender- and age-matched healthy controls (HCs). Patients were recruited at the Mental Health Center of West China Hospital, Sichuan University. Diagnosis was based on the Structured Clinical Interview (SCID) for DSM-IV and no patients with MDD had current comorbid Axis I or II diagnosis (such as psychosis, substance abuse, and personality disorder). In addition, patients who had taken any antidepressants during the past 3 months before scanning were excluded. At the time of the study, 61 patients were drug-naive and 30 had been medication-free for at least 3 months. HCs were recruited by advertisements in local communities. HCs without a lifetime history of any DSM-IV psychiatric disorder were recruited and not taking any antipsychotics or antidepressants. Furthermore, HCs with a family history of psychiatric disorders were excluded. Written informed consent was given by all participants after the study procedure had been fully explained. Exclusion criteria of patients and HCs were as follows: pregnancy, neurological or internal systemic diseases, a history of acute physical illness, a history of head injury resulting in loss of consciousness, and major neurological disorders, cardiovascular disease, intellectual disability, alcohol or substance abuse, contraindications to MRI, and a previous history of electroconvulsive therapy within the last 6 months. All participants were Han Chinese between the ages of 18 and 53 years.

### Clinical Assessment and Subgroups

Data were collected on age, gender, education level, the number of episodes, age of onset, and illness duration of all participants from their medical record. Clinical symptoms were assessed using the 17-item Hamilton Rating Scale for Depression (HDRS), which provided a total score and could be divided into five factors (anxiety/somatization, weight, cognitive disturbance, retardation, and sleep disturbance) (50). All patients were experiencing a major depressive episode, with their HDRS total scores being at least 17 on the day of scanning. We divided all patients into two subgroups: the EOD group (18–25 years old) and the LOD group (26–53 years old). HCs were also divided into two subgroups (young HCs and old HCs) matching for each patient subgroup. The onset age is defined by the age at which depressive symptoms firstly presented according to patients' recalls. The symptoms meet the diagnostic criteria for major depressive episodes in the Diagnostic and Statistical Manual of Mental Disorders IV (DSM-IV).

### Magnetic Resonance Imaging Procedure

All magnetic resonance imaging (MRI) data were acquired on a Philips 3.0-T scanner (Achieva TX, Best, the Netherlands), equipped with an eight-channel phased-array head coil. Gradient-echo-planar imaging sequence was applied to collect the rs-fMRI data with the following parameters: time repetition (TR) = 2,000 ms, echo time (TE) = 30 ms, flip angle = 90°, 38 slices, in-plane matrix =64 × 64, field of view (FOV) = 240 × 240 mm^2^, and voxel size = 3.75 × 3.75 × 4 mm^3^. For each participant, the rs-fMRI scanning lasted for 480 s, and 240 volumes were obtained. During the scanning, participants were fitted with soft ear plugs and instructed to relax with their eyes closed, and stay awake without moving.

To spatially normalize and localize better, high-resolution T1 images were acquired by 3D magnetization-prepared rapid gradient-echo sequence as follows: TR = 8.37 ms, TE = 3.88 ms, flip angle = 7°, matrix = 256 × 256, FOV = 240 × 240 mm^2^, voxel size =1.00 × 1.00 × 1 mm^3^, and 188 slices.

### Resting-State Functional Imaging Preprocessing

These rs-fMRI images were analyzed using the Data Processing Assistant for Resting-State fMRI (DPARSF, http://rfmri.org/DPARSF) (51) and SPM8 (http://www.fil.ion.ucl.ac.uk/spm), which is implemented in Matlab 2016a (MathWorks, Natick, MA, USA). First, the first 10 volumes were discarded, and functional volumes were slice-time corrected and head motion corrected. All participants in this study had <2 mm displacement and 2° of rotation in any direction. Second, the EPI images were registered to the T1 image, and then spatial normalization was performed to a 3 × 3 × 3 mm^3^ Montreal Neurological Institute template in SPM8. Third, the linear systematic drift of resulting data was removed and filtered at 0.01–0.08Hz frequency spectrum at each voxel. Finally, mean signal of white matter (WM), cerebrospinal fluid (CSF), and Friston 24-parameter model of head motion were regressed out.

### Regional Homogeneity

Individual ReHo maps were generated by calculating the Kendall's coefficient concordance of the time series of a given voxel with those of its nearest neighbors (26 voxels) in a voxel-wise manner (41). To reduce the effect of individual variations on the Kendall's coefficient of concordance value, ReHo maps were normalized by dividing the averaged Kendall's coefficient of concordance among each voxel of the whole brain. ReHo maps were smoothed with a 6-mm full-width half-maximum isotropic Gaussian kernel.

### Statistical Analysis

We compared the demographic and clinical data using the independent two-sample *t*-test, Chi-square test, or analysis of variance (ANOVA) between subgroups when appropriate, in Statistical package for Social Sciences (SPSS 22.0 for Windows). The significance threshold was set at *p* < 0.05.

Because the abnormal activation characteristic/pattern of brain often results from disease (depression), onset-age factor, and their interaction, a 2 × 2 full factorial model was used in SPM8, assessing the effects of onset age (young vs. old) and diagnosis (MDD vs. HCs) with age, gender, and education level as covariates; the interaction between onset age and diagnosis and the main effects of age-onset and diagnosis were tested. Multiple comparisons were corrected using the Monte Carlo simulation-based Alphasim program (1,000 times): the initial threshold on voxel level was set as *p* < 0.001, the cluster level was *p* < 0.05, and the corresponding cluster size was >16 (connection radius = 5 mm for correction).

**Figure 1:**
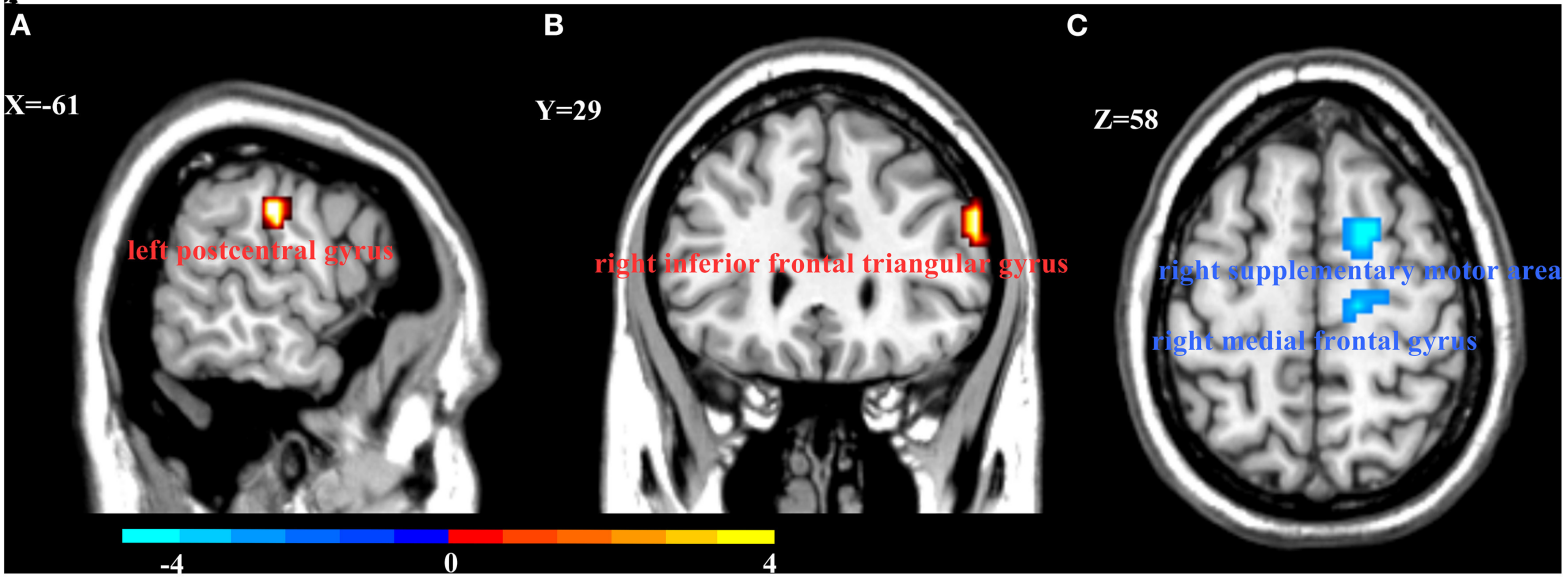
Interaction between onset age and depression on Regional homogeneity (ReHo). Compared with young HCs and LOD patients, EOD patients had increased ReHo in the left postcentral gyrus **(A)** and right inferior frontal triangular gyrus **(B)**. However, EOD patients had significantly decreased ReHo in the right superior frontal gyrus/supplementary motor area and the right medial frontal gyrus **(C)** than young HCs. Color bar represent the *t*-value.

*Post-hoc* analysis was conducted on extracted values of the clusters (Figure 1) that reached significance in the interaction analysis mentioned above. This analysis was completed in SPSS; Bonferroni-corrected pairwise comparisons were calculated to further investigate significant simple effects. To investigate the relationship between abnormal ReHo and clinical features, partial correlations were analyzed to explore the correlation between these abnormal values of ReHo and HDRS score or illness duration in each patient subgroup (age, gender, and years of education as covariates).

## Results

### Demographic and Clinical Characteristics of Participants

The study had 46 EOD patients, 45 LOD patients, 65 young HCs, and 50 old HCs. Patient subgroups did not differ significantly in gender, education years, number of episodes, HDRS scores, or illness course. Compared with each age-matched comparison group, no significant difference was observed in gender or age in the EOD and LOD groups except fewer education years in the old HC group. In addition, LOD patients have more severe sleep disturbance than EOD patients (Table 1).

**Table 1:** The demographics and clinical characteristics of the MDD and HCs subgroups.

**Variables (mean ± SD)**	**EOD**	**LOD**	**Young HCs**	**Old HCs**	***t*** **(***F***)/χ^2^**	***p***
Age (years)	20.3 ± 1.6	40.8 ± 5.3	21.6 ± 2.2	41.2 ± 6.8	344.05	<0.001^a^
Onset age (years)	18.7 ± 2.9	39.1 ± 4.8	/	/	−24.58	<0.001
Gender (male/female)	15/31	15/30	21/44	14/36	0.40	0.940^b^
Education (years)	13.7 ± 2.0	12.5 ± 3.5	14.4 ± 1.8	11.2 ± 4.0	12.41	<0.001^c^
Illness duration (months)	22.4 ± 29.9	20.4 ± 27.0	/	/	0.34	0.734
The number of episode	1.4 ± 1.0	1.6 ± 1.0	/	/	−1.10	0.274
HDRS total score	21.87 ± 4.08	23.32 ± 4.33	/	/	−1.64	0.104
Anxiety/somatization	5.54 ± 1.85	5.72 ± 1.82	/	/	−0.46	0.647
Weight	1.28 ± 0.89	1.38 ± 0.79	/	/	−0.56	0.574
Cognitive disturbance	4.30 ± 1.44	3.85 ± 1.41	/	/	1.51	0.136
Retardation	7.46 ± 1.70	8.04 ± 1.65	/	/	−1.67	0.099
Sleep disturbance	3.57 ± 1.41	4.32 ± 1.46	/	/	−2.52	0.013

*MDD, major depressive disorder; HCs, healthy controls; EOD, early-onset depression; LOD, late-onset depression; young HCs, young healthy controls; old HCs, old healthy controls; HDRS, hamilton rating scale for depression*.

a *The p-values were obtained by one-way analysis of variance tests. Post-hoc t-test (Bonferroni corrected): p = 0.161 (EOD vs. young HCs) and p = 0.712 (LOD vs. older HCs)*.

b *The p-values for gender distribution among the four groups were obtained by chi-square test (Bonferroni corrected): p = 0.973 (young HCs vs. EOD), p = 0.573 (old HCs vs. LOD), and p = 0.941 (EOD vs. LOD)*.

c *The p-values were obtained by one-way analysis of variance tests. Post-hoc t-test (Bonferroni corrected): p = 0.215 (EOD vs. young HCs), p = 0.026 (LOD vs. older HCs), and p = 0.064 (EOD vs. LOD)*.

### ReHo Alterations in MDD Patients and HCs

**Figure 2:**
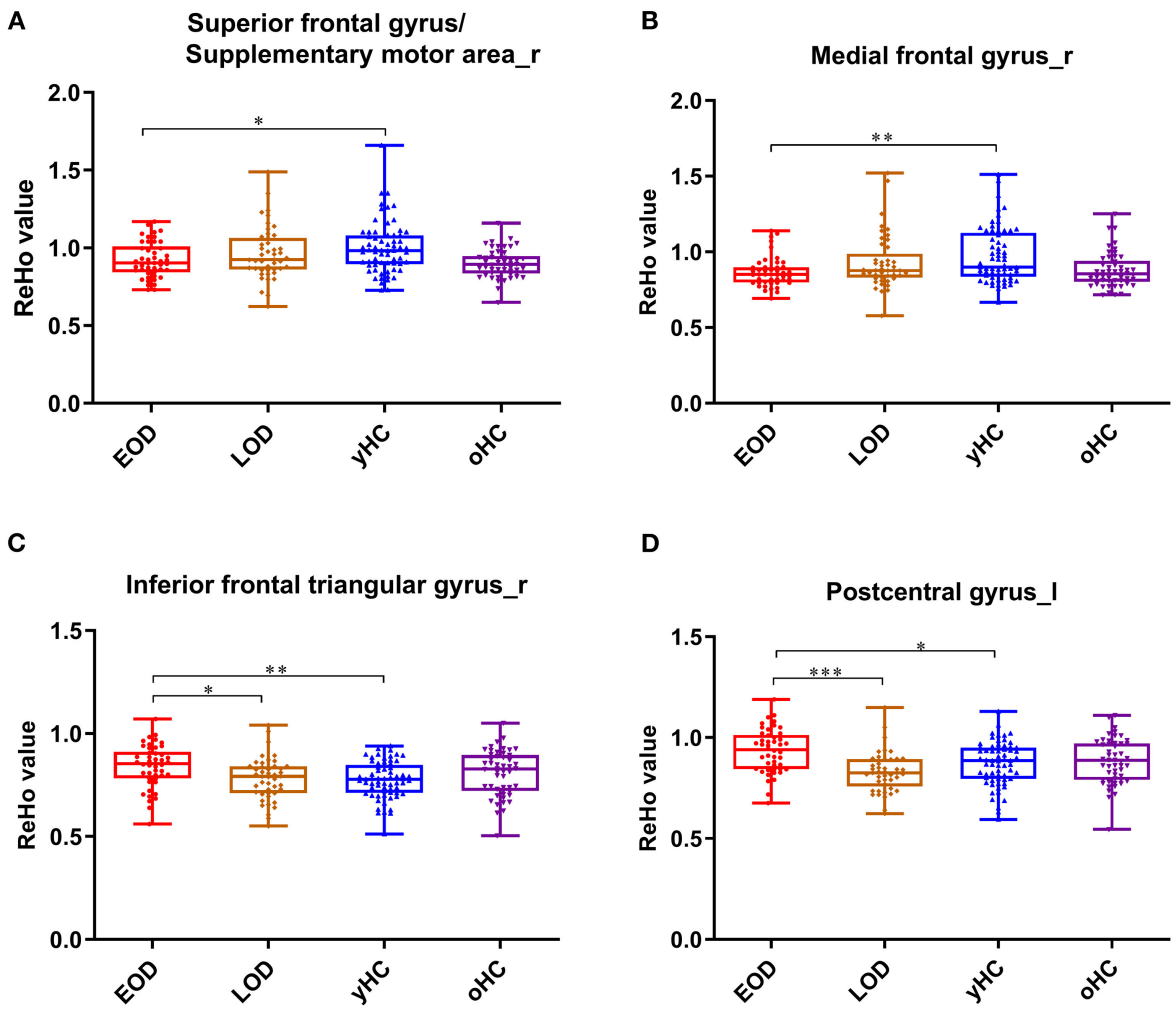
The box plots for the comparison between EOD, LOD patients, and young and old HCs. In the right superior frontal gyrus (supplementary motor area) **(A)**, right medial frontal gyrus **(B)**, right inferior frontal triangular gyrus **(C)**, and lest postcentral gyrus **(D)**. ^*^p < 0.05, ^**^p < 0.01, ^***^p < 0.001.

There was a significant interaction between onset age and depression in the inferior frontal triangular gyrus, postcentral gyrus, superior frontal gyrus (supplementary motor area), and medial frontal gyrus. Further *post-hoc* analysis showed that compared with LOD patients and young HC, EOD patients had significantly increased ReHo in the right inferior frontal triangular gyrus and the left postcentral gyrus. However, compared with young HC, EOD patients showed decreased ReHo in the right superior frontal gyrus/supplementary motor area and the right medial frontal gyrus (Table 2; Figures 1, 2). The old HCs and LOD patients did not differ significantly in ReHo.

**Table 2:** Regions showing altered ReHo among the EOD and LOD patients and HCs.

**Brain region**	**Side**	**Cluster size (voxels)**	**Coordinate** **(***X***, ***Y***, ***Z***)**			**BA**	***t-*** **Value**
**Interaction between diagnosis and onset age**							
**EOD > LOD**							
Inferior frontal triangular gyrus	R	16	57	30	27	46	4.07
Postcentral gyrus	L	20	−63	−18	30	1	4.03
**EOD > Young HC**							
Inferior frontal triangular gyrus	R	16	57	30	27	46	4.07
Postcentral gyrus	L	20	−63	−18	30	1	4.03
**EOD < Young HC**							
Superior frontal gyrus/Supplementary motor area	R	30	15	6	60	6	−4.14
Medial frontal gyrus	R	18	15	−18	60	6	−3.63
**Main effect of diagnosis MDD > HC**							
Middle frontal orbital gyrus	L	50	−39	48	−9	11	4.29
Superior temporal gyrus	R	30	72	−30	9	42	3.75
**MDD < HC**							
Precentral gyrus	L	25	−39	3	33	9	−4.19
Middle cingulum area	L	46	−9	−21	45	31	−3.96
**Main effect of age of onset Young < Old**							
Inferior temporal gyrus	L	26	−54	−15	−39	20	−4.19
**Young > Old**							
Supplementary motor area	R	33	6	3	72	6	4.26

*ReHo, regional homogeneity; L, left; R, right; BA, Brodmann area; EOD, early-onset depression; LOD, late-onset depression; MDD, major depressive disorder; HCs, healthy controls. The direction of post-hoc analysis results for interaction between onset age and depression obtained from Figure 2*.

**Figure 3:**
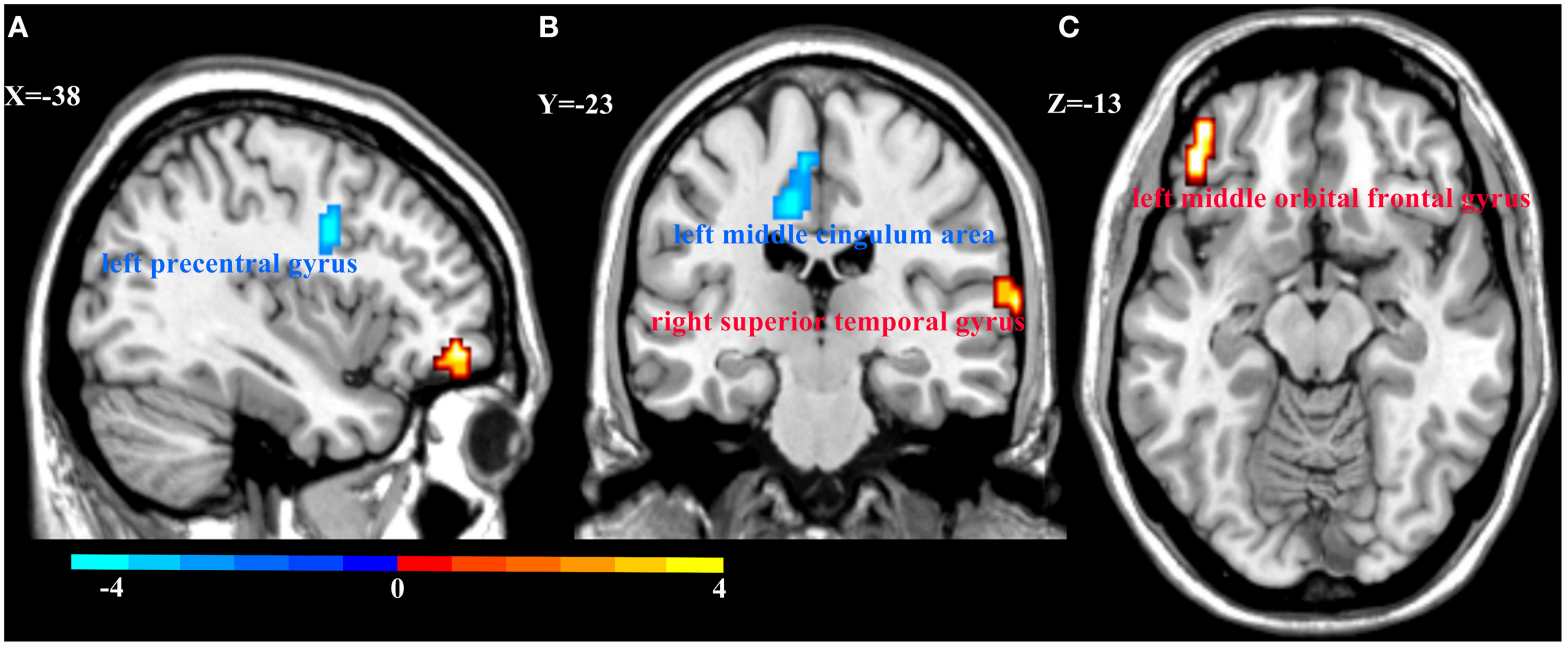
Regional homogeneity (ReHo) alterations in MDD patients and HCs. Compared with HCs, patients with MDD showed decreased ReHo in the left precentral gyrus **(A)** and left middle cingulum area **(B)**, and increased ReHo in the right superior temporal gyrus **(B)** and left middle orbital frontal gyrus **(C)**. Color bar represent the *t*-value.

We also explored the main effects of onset age and depression separately (Table 2; Figure 3). We focused on the main effects of depression here; compared with HCs, MDD patients showed decreased ReHo in the left precentral gyrus and left middle cingulum area, and increased ReHo in the left middle orbital frontal gyrus and right superior temporal gyrus.

### Correlation Between ReHo and Clinical Symptoms

**Figure 4:**
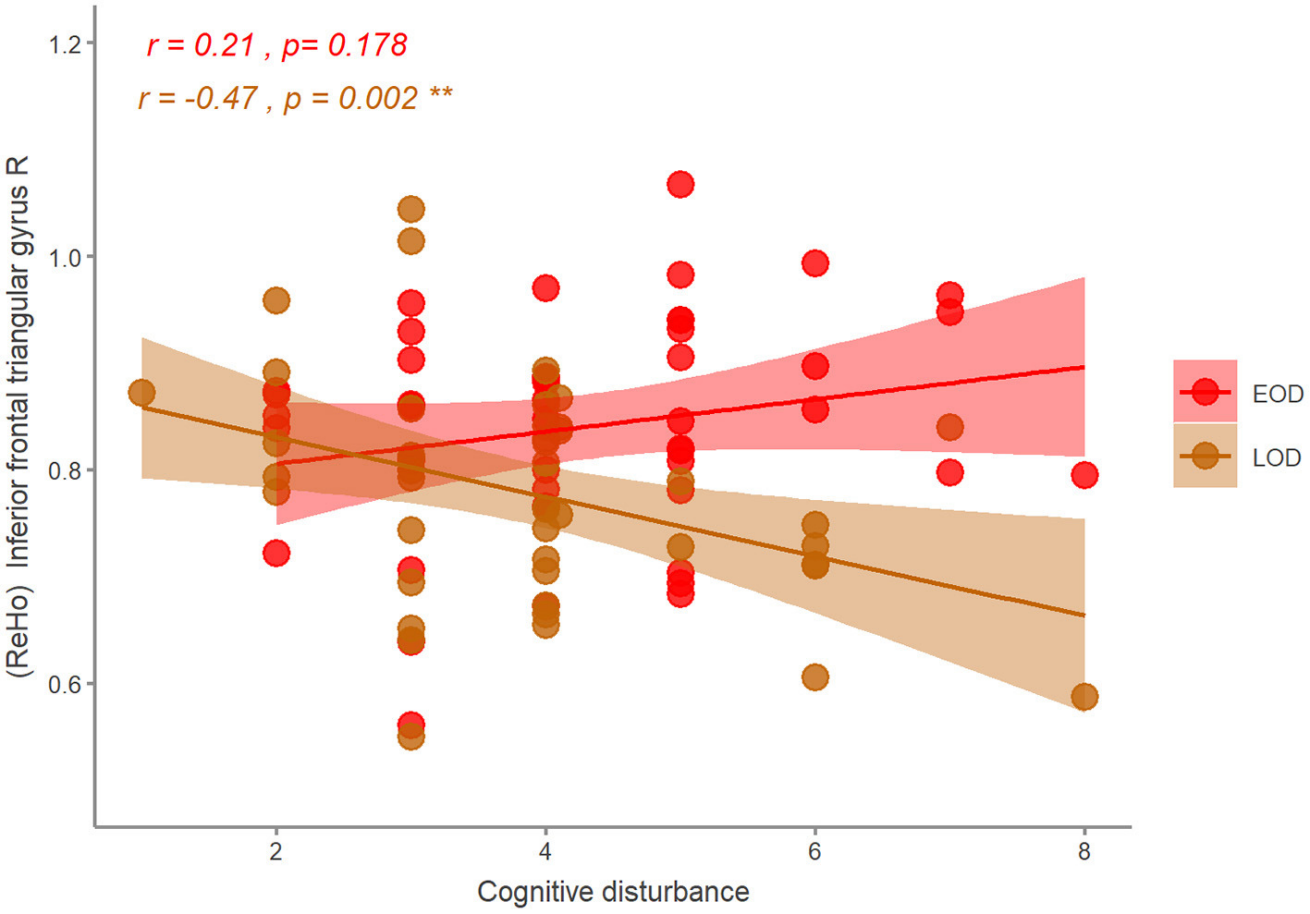
The correlation of the ReHo and HDRS symptom in EOD and LOD patients. EOD, early-onset depression; LOD, late-onset depression. ^**^p < 0.01.

With age, gender, and years of education as covariates, we found that ReHo in the right inferior frontal triangular gyrus was negatively correlated with the severity of cognitive disturbance in LOD patients (*r* = −0.47, *p* = 0.002). However, ReHo in the right inferior frontal triangular gyrus was positively correlated with the severity of cognitive disturbance in EOD patients, but not significant (*r* = 0.21, *p* = 0.178) (Figure 4). No other significant correlations were detected (Table 3).

**Table 3:** The correlation of the ReHo and HDRS symptom in patients with MDD.

	**HDRS**	**Anxiety/somatization**	**Weight**	**Cognitive disturbance**	**Retardation**	**Sleep disturbance**
**EOD:** ***r*** **(***p***)**						
Inferior frontal triangular gyrus R	0.24 (0.116)	0.23 (0.131)	0.22 (0.150)	0.21 (0.178)	0.17 (0.278)	0.11 (0.477)
Postcentral gyrus L	0.003 (0.987)	−0.03 (0.835)	−0.18 (0.253)	0.16 (0.318)	0.10 (0.512)	−0.07 (0.663)
**LOD:** ***r*** **(*****p*****)**						
Inferior frontal triangular gyrus R	−0.23 (0.151)	−0.01 (0.977)	−0.17 (0.286)	−0.47^**^ (0.002)	−0.11 (0.505)	−0.02 (0.906)
Postcentral gyrus L	−0.21 (0.180)	−0.18 (0.263)	0.10 (0.551)	−0.14 (0.375)	−0.29 (0.065)	0.0004 (0.998)

*MDD, major depressive disorder; EOD, early-onset depression; LOD, late-onset depression; ReHo, regional homogeneity; HDRS, Hamilton Rating Scale for Depression*.

** *p < 0.01*.

## Discussion

The present study is the first large-scale clinical study to examine the characteristics of medication-free MDD patients with different onset age by combining clinical presentation and neuroimaging phenotypes. Our study indicated that EOD patients were associated with greater disturbances in ReHo than LOD patients, and this disturbance was related to the symptoms of different onset-age patients. The present study provides novel insights into how development of depression is depicted by different onset age.

At the neuroimaging level, MDD patients showed decreased ReHo in the left precentral gyrus and the left middle cingulum area, and increased ReHo in the left middle orbital frontal gyrus and right superior temporal gyrus than HCs. These results may provide some evidence for the impairment of reward processing in MDD patients, consistent with previous studies. On the one hand, OFC is involved in non-reward and punishing events, and has increased functional connectivity with self-referential processes (medial prefrontal cortex, precuneus, and posterior cingulate cortex) causing loss, depression, and frustration (52–54). The increased ReHo of the right superior temporal gyrus lay in the auditory processing network, which may contribute to perceptual impairments in MDD patients (44, 45). On the other hand, cingulate gyrus dysfunction affects the release of dopamine and induces cognitive impairment, which mainly manifests with slow thinking and cognitive dysfunction in MDD patients. Bluhm et al. (55) reported that MDD patients had decreased connectivity between the PCC and the bilateral caudate, regions known to be involved in motivation and reward processing, which is characterized by lack of motivation and loss of pleasure and may be an early manifestation of MDD.

Further results showed that compared with LOD patients and young HCs, EOD patients had significantly increased ReHo in the right inferior frontal triangular gyrus and the left postcentral gyrus. The right inferior frontal triangular gyrus is an important component of the dorsolateral prefrontal cortex (DLPFC). The DLPFC dysfunction may be an early manifestation of MDD and that the right inferior frontal triangular gyrus plays an important role in the pathogenesis of the early stage of MDD; patients with mild DLPFC damage often appear to be uninterested in things, with slow thinking, poor memory, and lack of motivation (56–59). Recently, one study using machine learning methods reported the abnormal ReHo in the left postcentral gyrus and right fusiform gyrus with a high accuracy for discriminating MDD from HC (60). These results and our study provide further evidence for the important role of the prefrontal lobe in MDD, which may help distinguish the depression subtypes for accurate diagnosis and treatment in the future.

Our study found that compared with young HCs, EOD patients showed decreased ReHo in the right superior frontal gyrus/supplementary motor area and the right medial frontal gyrus. On the one hand, this trend of group difference was consistent with Guo et al. (61), who found that, using ALFF indicator, EOD patients had abnormal brain activation in the left superior/inferior temporal gyrus, left lingual gyrus, and right middle occipital gyrus compared to young HCs. On the other hand, these abnormal brain areas of EOD patients are mainly concentrated in the prefrontal gyrus (PFC) (39). The PFC is the executive control center for information processing, responsible for the processing of functional cognitive processes and related to the control of emotion and conflicting behaviors (62). Frontal hyperactivity has been viewed as an exaggerated or maladaptive compensatory process, serving to improve a persistent negative mood generated by abnormal chronic activity of limbic–subcortical structures. However, frontal hypo-metabolism may be associated with the failure to initiate or maintain a compensatory state (63), and then the depression became more serious. These studies suggest that the PFC may be the key area for the treatment of brain damage and disease in EOD patients. Moreover, our study found that compared with HCs, there were no significant changes in ReHo of LOD patients, which indicated that the degree of local function impairment is more serious in EOD patients than in LOD patients. The potential pathophysiological mechanisms of MDD patients at different onset age may be different.

Sleep disturbances are the major somatic symptoms in MDD patients (64), while LOD patients experience more severe sleep disturbances, especially early morning awakening. Sleep disturbance is a significant risk factor for the onset, exacerbation, and relapse of mood disorders (65). In addition to changes in sleep architecture, MDD patients show profoundly altered patterns of nocturnal hormone secretion, possibly through mechanisms that link regulation of sleep with neuroendocrine activity (66). Early relief of insomnia in MDD patients, in addition to alleviating other symptoms, may increase adherence to treatment and increase daytime performance and overall functioning, while complete relief of insomnia may improve prognosis (67). The difference on sleep disturbance between EOD and LOD patients may provide further evidence to distinguish these two subtypes of depression.

We found that ReHo in the right inferior frontal triangular gyrus was negatively correlated with the severity of cognitive disturbance in LOD patients. The inferior frontal triangular gyrus was located in the prefrontal lobe, close to the OFC and middle frontal gyrus, which was associated with cognitive control (68, 69). Orbitofrontal cortex plays an important role in decision-making, and decision-making impairment is associated with a susceptibility to suicidal symptoms (70), which is included in the cognitive disturbance. A previous study also reported the decreased ReHo in the right middle frontal gyrus was negatively correlated with depression symptoms in MDD patients (60). On the contrary with LOD patients, we found that the correlation between ReHo in the inferior frontal triangular gyrus and cognitive disturbance of EOD was positive (not significant). Although the ReHo in the right inferior frontal triangular gyrus of EOD was decreasing, it may not directly affect (or be affected by) cognition. A previous study has detected the older depressed patients with severer cognitive impairment (71). Some researchers found that the cortical thickness in the prefrontal cortex was correlated with cognitive impairment of LOD, but not EOD (72), similar to our results using ReHo indicator. This may indicate that the EOD and LOD subgroups have different cognitive impairment and relationship with ReHo in the prefrontal cortex. More studies are needed to clarify this in the future. The combination of clinical symptomology and brain imaging has again confirmed that the prefrontal lobe, especially the right inferior frontal triangular gyrus, is a key brain area to distinguish EOD patients from LOD patients.

However, our study still has some limitations. First, because the present study is a cross-sectional study, we could not exclude the possibility that some of our patients who manifested as having a depressive episode could be later diagnosed as having a bipolar disorder. Further follow-up study may be necessary to clarify the impact of prolonged depressive episodes on brain structure in the future. Second, as in other resting-state fMRI studies, inevitable physiological noises such as respiratory and cardiac fluctuations of the participants during scanning may influence the stability of resting-state fMRI signals. Although we have tried our best to correct such noises, their effects may have not been fully eliminated. In future studies, simultaneous cardiac recording may provide a rigorous correction. Last, there is no consensus on onset age to define EOD and LOD. However, the cutoff age in the present study has been used as a cutoff in previous research on mood disorders (15, 34, 37–39) and genetic association studies (40), and is associated with family history of MDD (35, 36).

## Conclusion

We recruited a group of medication-free patients with major depressive disorder in our analysis to minimize confounding factors due to medication use, which could better elucidate ReHo changes that are directly related to the disease itself. MDD patients with different onset ages may have different pathophysiological mechanisms, and EOD patients had more abnormal ReHo than LOD patients in the prefrontal lobe, especially the right inferior frontal triangular gyrus.

## Data Availability Statement

The raw data supporting the conclusions of this article will be made available by the authors, without undue reservation.

## Ethics Statement

The studies involving human participants were reviewed and approved by the Ethical Committee of Sichuan University. The patients/participants provided their written informed consent to participate in this study. Written informed consent was obtained from the individual(s) for the publication of any potentially identifiable images or data included in this article.

## Author Contributions

ZZ and YC wrote the main manuscript text. XM, WW, and TL conceived and designed the study. ZZ, YC, TL, WW, XY, YM, HY, WG, QW, and WD recruited the patients, administered assessment tools, and carried out data analysis. XM reviewed the manuscript. All authors contributed to the article and approved the submitted version.

## Funding

This research was partly funded by the National Natural Science Foundation of China (No. 82001432 and No.81671344), the China Postdoctoral Science Foundation (No. 2020TQ0213 and No. 2020M683319), and 1.3.5 project for disciplines of excellence, West China Hospital, Sichuan University.

## Conflict of Interest

The authors declare that the research was conducted in the absence of any commercial or financial relationships that could be construed as a potential conflict of interest.

## Publisher's Note

All claims expressed in this article are solely those of the authors and do not necessarily represent those of their affiliated organizations, or those of the publisher, the editors and the reviewers. Any product that may be evaluated in this article, or claim that may be made by its manufacturer, is not guaranteed or endorsed by the publisher.
